# PERFECTA Loaded Lipid Cubosomes for ^19^F‐MRI: Physico‐Chemical Characterization

**DOI:** 10.1002/chem.71231

**Published:** 2026-06-08

**Authors:** Pietro Milesi, Beatrice Lucia Bona, Cristina Chirizzi, Marco Campanile, Pompea Del Vecchio, Marc Schmutz, Valerie Sartor, Debora Berti, Pierangelo Metrangolo, Francesca Baldelli Bombelli, Barbara Lonetti

**Affiliations:** ^1^ Department of Chemistry Materials and Chemical Engineering “Giulio Natta“ Milano Italy; ^2^ Neuroradiology Unit Fondazione IRCCS Istituto Neurologico Carlo Besta Milan Italy; ^3^ Department of Chemical Sciences University Federico II of Naples Napoli Italy; ^4^ CNRS Institut Charles Sadron Université de Strasbourg Strasbourg France; ^5^ Laboratoire Softmat Université de Toulouse Toulouse France; ^6^ Department of Chemistry, Ugo Schiff University of Florence Sesto Fiorentino Florence Italy

**Keywords:** ^19^F NMR/MRI, lipid self‐assembly, liquid crystalline nanoparticles, PERFECTA

## Abstract

Liquid crystalline nanoparticles (LCNPs), such as cubosomes, are promising platforms for drug delivery and bioimaging due to their unique internal structures and biocompatibility. Here, we developed phytantriol‐based cubosomes stabilized with Pluronic F‐127 and loaded with PERFECTA, a fluorinated probe with 36 magnetically equivalent fluorine atoms, for use as ^19^F‐MRI nanoprobes. PERFECTA loaded cubosomes were synthesized via solvent evaporation and characterized using DLS, SAXS, Cryo‐TEM, DSC, ^19^F ‐NMR, and ^19^F ‐MRI. Results show that PERFECTA‐loaded cubosomes retain the Pn3m cubic phase, achieving encapsulation efficiencies over 70% across PERFECTA concentrations (1–10 mg/mL). Cryo‐TEM revealed PERFECTA segregation into spherical domains, either embedded within cubosomes or as isolated nanoparticles. SAXS and DSC analyses suggested a partial PERFECTA integration into the phytantriol bilayer, affecting the internal liquid‐crystalline order. PERFECTA loaded cubosomes demonstrated excellent colloidal stability in biologically relevant media, maintaining structural integrity and fluorine content within 24 h in cell culture medium with fetal bovine serum. Thanks to their favorable relaxometric properties (T_1_ = 497 ± 50 ms, T_2_ = 281 ± 4 ms) and robust ^19^F MRI signals, these cubosomes hold significant potential as stable, efficient nanotheranostic platforms for biomedical imaging and drug delivery.

## Graphical abstract

Phytantriol‐based cubosomes loaded with PERFCTA, a fluorinated MRI probe, were developed as promising systems for imaging and drug delivery. The nanoparticles showed high PERFECTA encapsulation efficiency, strong fluorine MRI signals, and optimal colloidal stability in biological media. Their favorable imaging properties and stability highlight their potential as effective nanotheranostic platforms for biomedical applications.

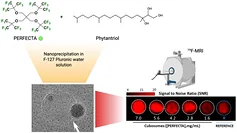

## 1 Introduction

Lipid self‐assembly in water is driven by hydrophobic effect, resulting from a subtle balance between hydrophobic tails and hydrophilic headgroups and can produce different structures in the nanoscale such as micelles, liposomes, bicontinuous liquid crystalline (LC) phases and nanoparticles (NPs). Lipid‐based self‐assemblies were among the first nanosystems to be developed for drug delivery and receive clinical approval [1]. Owing to their inherent biocompatibility and straightforward synthesis, lipids are a key component in the majority of FDA‐approved nanoformulations [2, 3]. Furthermore, their remarkable versatility was underscored during the COVID‐19 pandemic, when major regulatory agencies authorized the use of lipid‐based formulations (LNPs) for messenger RNA (mRNA) vaccines [4].

An emerging class of lipid nanosystems for drug/gene delivery are liquid crystalline nanoparticles (LCNP), such as hexosomes and cubosomes. These self‐assemblies are distinguished by their intricate nonlamellar internal architectures, including two‐ and three‐dimensional structures like inverse hexagonal (hexosomes) and cubic (cubosomes) phases [5]. In particular, cubosomes are formed by highly curved lipid bilayers, which self‐assemble into a complex 3D structure with cubic lattices, where lipid hydrophobic regions coexist with aqueous channels. Given this complex structure, compared to liposomes, cubosomes possess multiple advantages. They possess high membrane surface area to volume ratios (400 m^2^/g) [6], which could significantly improve loading of hydrophobic compounds like drugs or imaging probes. Moreover, the presence of interwoven channels within the structure could allow for hydrophilic molecules loading, with tuneable drug release ratios. Finally, recent studies have highlighted the fusogenicity of cubosomes, potentially facilitating their cellular uptake [7, 8, 9]. Cubosomes are typically formed using glyceryl monooleate (GMO) or phytantriol as primary structural lipids, while steric stabilizers such as Pluronic, a triblock copolymer composed by a central hydrophobic polypropylene oxide (PPO) block surrounded by two hydrophilic polyethylene oxide (PEO), are added to guarantee NP colloidal stability and monodispersity. Stabilizers play a crucial role in the fate of cubic lipid NPs, especially tuning their interactions with cells and biological environments. Thermo‐responsive block copolymers were recently tested to enhance cubosomes affinity toward lipid bilayer membranes and better control release of the encapsulated molecules [10, 11, 12].

**FIGURE 1:**
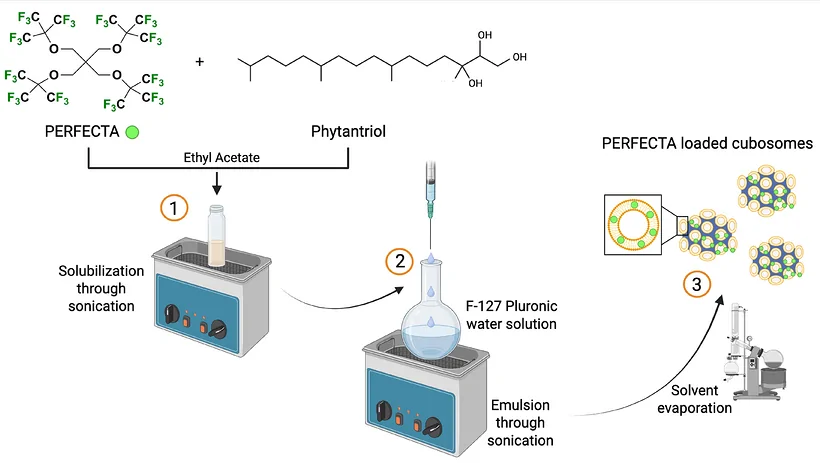
Schematic drawing of the protocol used for prepare PERFECTA‐loaded cubosomes based on phytantriol and F‐127 pluronic. Created in BioRender, https://BioRender.com/hic3o9r.

Unlike most studies [9, 10, 11], where cubosomes were loaded with fluorescent dyes or nitroxide lipids for in vitro and/or in vivo imaging, we developed a strategy to produce cubic LCNP, based on phytantriol and Pluronic F‐127, capable of incorporating the fluorine19 magnetic resonance imaging (^19^F‐MRI) probe PERFECTA (Figure 1) [13, 14], thereby imparting intrinsic imaging capability to the system, while leveraging their high fusogenicity to enhance internalization within cells. In fact, MRI stands out as a particularly powerful analytical tool, owing to its non‐invasive nature, lack of ionizing radiation, and excellent tissue penetration. In this context, ^19^F‐MRI has emerged as a highly promising technique for cell tracking, as fluorinated probes can be detected with virtually no endogenous background signal, enabling unambiguous localization in vivo. Notably, the ^19^F‐MRI signal intensity scales linearly with the number of fluorine atoms, making probe design crucial for sensitivity enhancement. Therefore, probes with a high density of magnetically equivalent ^19^F nuclei are especially desirable. In this regard, the compound PERFECTA was selected, as it contains 36 magnetically equivalent fluorine atoms and has already demonstrated ability to function as dual imaging probe for ^19^F‐MRI and Raman microscopy [15]. A combination of complementary analytical techniques was employed to assess probe encapsulation efficiency, its impact on the nanostructure and ^19^F‐MRI performance. Specifically, Dynamic Light Scattering (DLS), Small‐Angle X‐ray Scattering (SAXS), Cryogenic Transmission Electron Microscopy (Cryo‐TEM), Differential Scanning Calorimetry (DSC), Nuclear Magnetic Resonance (NMR), and ^19^F‐MRI were used to comprehensively characterize the obtained NPs. Attention was devoted to elucidate the internal organization of the fluorinated probe within the self‐assembled phytantriol/Pluronic matrix, as a function of PERFECTA loading. This approach enabled us to correlate structural features with probe incorporation, providing insights into the distribution and accommodation of PERFECTA within the cubic lipidic framework [15, 16, 17].

## 2 Experimental Section

### 2.1 Materials

Phytantriol (mixed isomers, C_20_H_42_O_3_, purity over 95%) was purchased from TCI. Pluronic 127 ((C_3_H_6_O.C_2_H_4_O)_x_) was purchased from Sigma Aldrich (Merck). PERFECTA was synthesized according to protocol already published [13], ultrapure type – I Milli‐Q Water (18.2 MΩ cm, organic carbon content ≥ 4 µg L^−1^).) was provided by a Simplicity water purification system. All chemicals and reagents were at the analytical grade and used as received from supplier.

### 2.2 Methods

#### 2.2.1 PERFECTA Loaded Cubosomes Preparation

PERFECTA loaded cubosomes were prepared using the solvent evaporation method by dissolving phytantriol (50 mg) and PERFECTA (in the range 2 to 20 mg) in ethyl acetate (1 mL). The mixture was vortexed for 5 min and sonicated in a bath (59 kHz, 70°C) for 10 min. The obtained organic phase was dropwise added to an aqueous solution (2 mL) containing F‐127 Pluronic (17% or 35% w/w with respect to phytantriol mass). The mixture was emulsified by bath sonication (70°C, 59 kHz of frequency, 30 min), and subsequently ethyl acetate was removed by evaporation.

#### 2.2.2 ^19^F‐Nuclear Magnetic Resonance (NMR)

^19^F‐NMR experiments were performed using a Bruker AV400 spectrometer. Analyses were performed at room temperature and chemical shifts were reported in part per million (ppm). Encapsulation efficiency (EE%) of PERFECTA was determined by quantitative ^19^F‐NMR experiments using trifluoroacetic acid ‐TFA (chemical shift at −75.5 ppm)‐ as an external standard at a known concentration. The amount of encapsulated PERFECTA was calculated as a ratio between the integrated areas of the signals of PERFECTA and TFA, as the signal is directly proportional to the number of fluorine nuclei. The obtained value was then converted into concentration of fluorine atoms per mL and used to calculate the EE% (see equation below) relative to the initially added PERFECTA concentration.

$$\mathrm{EE}\%=\frac{\left({\mathrm{PERFECTA}}_{\mathrm{encapsulated}}\right)}{\left({\mathrm{PERFECTA}}_{\mathrm{added}}\right)}\times 100$$

^19^F longitudinal (*T*_1_) and transverse (*T*_2_) relaxation times measurements were recorded at 305 K on a Bruker AV400 spectrometer operating at 400 MHz for the ^1^H nucleus. The inversion recovery and the CPMG pulse sequences were used for the measurements of *T*_1_ and *T*_2_, respectively.

#### 2.2.3 ^19^F‐ Magnetic Resonance Imaging (MRI)

^1^ ^9^F‐MRI analysis was performed on cubosome formulations prepared at five different PERFECTA concentrations, (1.6–7.0 mg/mL) in 150 µL of Milli‐Q water. Each sample was loaded into a 0.2 mL Eppendorf tube and placed in a phantom filled with 2% agar gel. All experiments were carried out on a 7 T scanner (BioSpec, Bruker BioSpin) equipped with a dual‐transmit/receive ^1^ ^9^F/^1^H volume coil (35 × 59 mm) and an ultra‐shielded horizontal bore magnet. ^1^ ^9^F‐MR images were acquired at the PERFECTA‐specific resonance frequency (−73.4 ppm). A 3D turbo spin‐echo sequence was used for both ^1^H and ^1^ ^9^F acquisitions with the same field of view (45 × 30 × 24 mm), using TR/TE = 250/15 ms, matrix = 128 × 64 × 8, and 6 averages for ^1^H‐MRI, and TR/TE = 1600/40 ms, matrix = 64 × 32 × 8, and 80 averages for ^1^ ^9^F‐MRI. For each sample, the signal‐to‐noise ratio (SNR) was measured using the scanner image analysis software (ParaVision 6.0, Bruker BioSpin). Mean signal intensity was measured within the sample region on the ^1^ ^9^F image and divided by the standard deviation of the noise, calculated from at least three regions outside the samples. The total number of ^1^ ^9^F atoms was estimated relative to the SNR of a PERFECTA reference standard consisting of a lecithin emulsion containing 2.3 mg/mL PERFECTA.

#### 2.2.4 Dynamic Light Scattering (DLS)

DLS measurements were performed on an ALV apparatus equipped with ALV‐5000/EPP Correlator, special optical fiber detector and ALV/CGS‐3 Compact goniometer (ALV‐GmbH, Langen/‐ Germany). The light source is He–Ne laser (*λ* = 633 nm), 22 mW output power. Measurements were performed at 25°C. Approximately 1 mL of 1:50 NPs sample dispersion in MilliQ water or in 10% FBS – DMEM culture media (without phenol red) was transferred into the cylindrical Hellma scattering cell. Data analysis was performed according to standard procedures and auto‐correlation functions – measured at 90° scattering angle (θ) – were analysed through both cumulant analysis and a constrained regularization method, CONTIN, for obtaining both z‐averaged hydrodynamic radius (and PDI) and NPs intensity weighted hydrodynamic radius (R_H_) distribution. Viscosity for 10% FBS – DMEM medium was set to 1.20 mPa s. The choice to report data at *θ* = 90° was related to the low angular dependence of the analysis at this scattering angle compared to, for example, backscatter analysis, where larger components are not visible. Analysis at *θ* = 90° thus allows for obtaining a more complete understanding of the analysed sample. Polydispersity indexes (PDI) were obtained by cumulant fitting. Each measurement was repeated three times, and for each sample three batches were independently measured. Standard deviations were calculated accordingly.

#### 2.2.5 Small‐Angle X‐Ray Scattering (SAXS)

The SAXS measurements were performed with a Xeuss 3.0 UHR SAXS/WAXS system (Xenocs SAS, Grenoble, France) equipped with a GeniX 3D Cu K alpha radiation source (*λ* ≈ 1.54 Å) and with in‐vacuum motorized 3‐axis Q‐Xoom Eiger2 R 1M detector (Dectris Ltd., Switzerland). The configuration chosen for the measurements covered a q range of 0.007 < q (Å^−1^) < 0.5 Å^−1^, where *q = 4π / λ* sin*(θ/2)*, *q* is the modulus of the scattering vector, λ the wavelength, and θ the scattering angle. The samples were measured in quartz capillary of 1.5 mm diameter at RT and under vacuum (< 0.09 mbar) for 3 h. The 2D SAXS patterns were radially integrated into the one‐dimensional scattering intensity *I(q)* and merged. The data presented are background subtracted, where the background corresponds to the solvent measured in an analogue capillary.

#### 2.2.6 Cryo‐Transmission Electron Microscopy (TEM)

A volume of 5 µL was applied on freshly glow‐discharged 400 mesh copper TEM grids covered with a lacey carbon film. The grids were blotted and immersed into liquid ethane cooled by liquid nitrogen using a homemade freezing machine equipped with a controlled temperature and humidity chamber (set at 22°C and > 80% relative humidity). The grids were then mounted onto a Gatan 626 cryoholder and observed under low dose conditions using a Tecnai G2 microscope (FEI) operating at 200 kV. The images were recorded with a slow‐scan CCD camera (Eagle 2k2k, FEI).

#### 2.2.7 Differential Scanning Calorimetry (DSC)

Differential scanning calorimetry (DSC) measurements were carried out using a nano‐DSC (TA Instruments, New Castle, DE, USA) equipped with twin gold capillary cells (300 µL) at a constant pressure of 3 atm. The sample suspension and water were loaded into the calorimetric and reference cells, respectively. Each sample was subjected to a thermal scan from 10 to 70°C at the scanning rate of 1°C min^−1^. The resulting thermograms were analyzed using NanoAnalyze software provided by the manufacturer and processed for presentation with Origin software (OriginLab, Northampton, MA, USA).

## 3 Results and Discussion

Phytantriol is one of the most common lipids used to formulate cubosomes and was chosen for its high stability to sonication and temperature treatment. Building on our previous experience in developing PERFECTA formulations, we adapted an established emulsification protocol [14] to assess its suitability for the formation of phytantriol‐based cubosomes, with the aim of subsequently incorporating PERFECTA. Using ethyl acetate as organic solvent (see Methods), this approach successfully yielded *Pn3m* cubic‐phase NPs with a lattice parameter of 95.5 ± 0.5 Å (see Figure S1 and Table S1). The same procedure was then applied to obtain PERFECTA loaded cubosomes, setting 35% (w/w) as the minimal concentration of the stabilizer, F‐127, to obtain monodisperse NPs. Different formulations, varying PERFECTA initial concentration, were prepared and characterized by DLS and ^19^F‐NMR to evaluate their size distribution and PERFECTA encapsulation efficiency (EE) (Table 1), respectively. Results showed that both hydrodynamic size and polydispersity were not affected by the presence of PERFECTA. EE% were similar, with only a slight decrease at the highest concentration. The obtained formulations showed colloidal stability over two weeks from their preparation when stored at RT (See Figure S2).

**TABLE 1:** Properties of the different formulations varying PERFECTA initial concentration.

[PERFECTA]_added_ ^a^ (mg/mL)	d_H_ (nm) (SD)^b^	PDI (SD)^b^	PERFECTA EE (%, SD)^c^
0	229 (47)	0.15 (0.02)	—
1	203 (21)	0.13 (0.01)	81 (14)
5	235 (28)	0.11 (0.03)	77 (14)
10	221 (22)	0.15 (0.03)	73 (12)

^a^ Nominal PERFECTA concentration, expressed as weighted mass of PERFECTA per volume in the final mixture, used during the preparation of the dispersion.

^b^ d_H_ and PDI values were obtained by DLS experiments by cumulant analysis, SD derive from the mean of at least three different measurements.

^c^ Encapsulation efficiency is defined as the mass percentage of encapsulated PERFECTA with respect to the weighted one and evaluated through ^19^F NMR; SD provided as mean of at least three different measurements.

**FIGURE 2:**
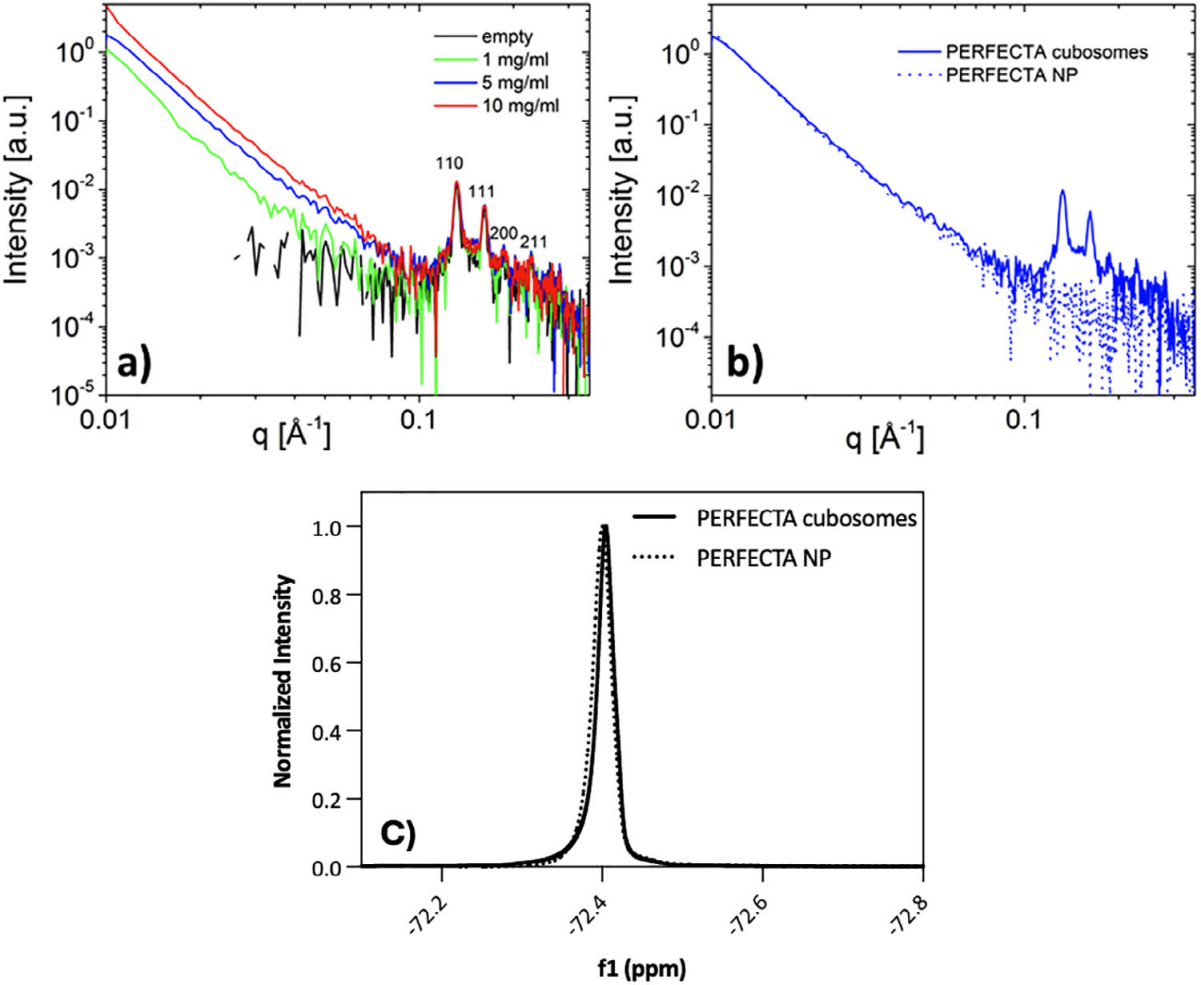
(a) SAXS intensity as a function of the scattering vector, comparison between empty and PERFECTA loaded cubosomes; (b) comparison between PERFECTA cubosomes and F‐127 PERFECTA NPs at PERFECTA concentration of 5 mg/ml; (c) ^19^F‐NMR spectra of PERFECTA loaded cubosomes and F‐127 NPs, focusing on PERFECTA peak. Color codes as reported in the legend of the figure.

The obtained PERFECTA formulations were then characterized by SAXS to verify the preservation of the liquid crystalline phase upon PERFECTA encapsulation. As shown in Figure 2a, the PERFECTA‐loaded assemblies retain the *Pn3m* cubic structure observed for empty cubosomes with lattice parameters remaining almost constant across all PERFECTA concentrations investigated (see Table S1). Moreover, a power‐law increase was observed in the low scattering vector (q) region, with the intensity scaling with PERFECTA concentration. We attributed this behaviour to the possible co‐presence of F‐127‐stabilized PERFECTA NPs [18]. To investigate this hypothesis, PERFECTA NPs were prepared using the same solvent evaporation method, but without phytantriol, the lipid responsible for inducing the cubic phase. As reported in Table S2, F‐127‐stabilized PERFECTA NPs were successfully formed with good EE% (60%–70%), although they exhibited both larger size and higher PDI (0.35 – 0.5). Comparison of SAXS profiles of PERFECTA loaded cubosomes and F‐127 PERFECTA NPs (Figure 2b, [PERFECTA] = 5 mg/ml) revealed overlapping curves in the low‐q region. Notably, the latter did not exhibit any diffraction peaks indicative of an internal liquid crystalline order. These observations supported the assignment of the low‐q scattering contribution to the presence of PERFECTA spherical domains also in the cubosome samples. ^19^F‐NMR measurements were also performed on both samples to evaluate potential chemical shift differences arising from distinct chemical environments. The spectra were nearly identical (Figure 2c), showing a single peak at about − 72.4 ppm, indicating a comparable local environment of PERFECTA in both systems.

**FIGURE 3:**
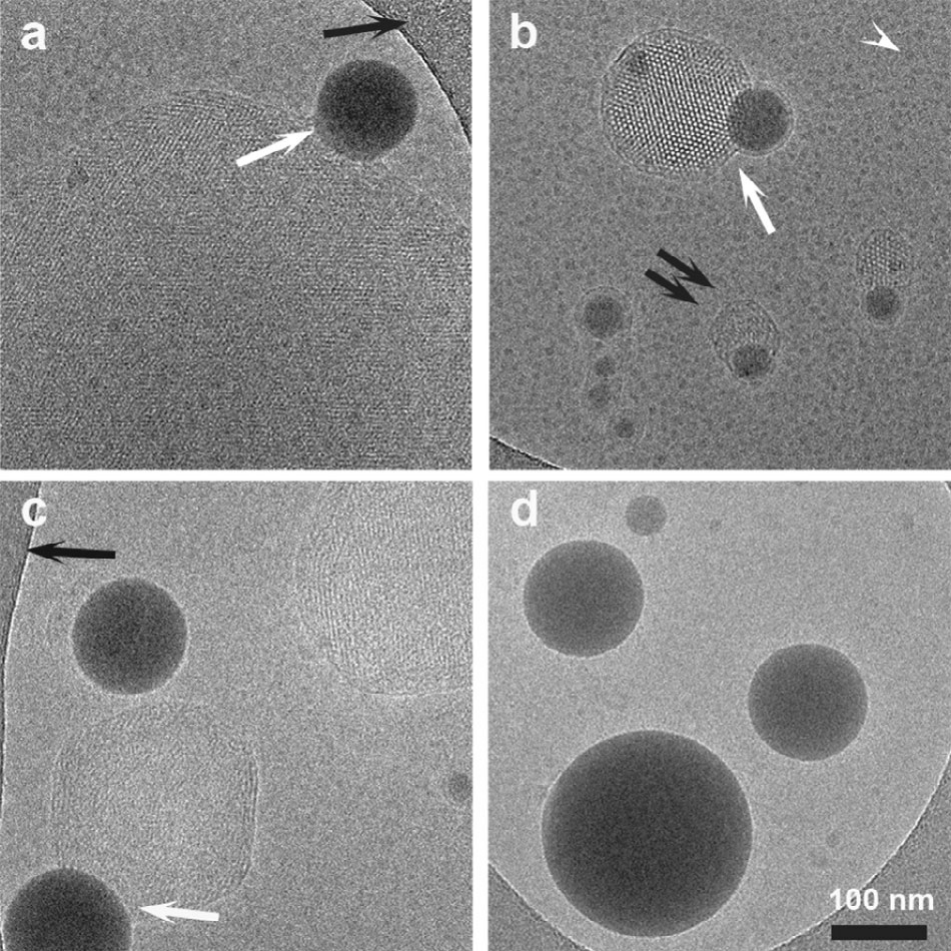
Cryo‐TEM images of phytantriol cubosomes stabilized by F127 prepared at different PERFECTA concentrations: (a) 1 mg/ml; (b) 5 mg/ml; (c) 10 mg/ml. Cryo‐TEM image of F127 PERFECTA NPs prepared at 10 mg/ml of PERFECTA (d). The black arrows indicate the supporting formvar/carbon film. The white arrowhead in b) points toward some water contamination. The white arrows point toward Perfecta nanoparticles attached to the cubosomes. The double black arrow shows some non‐structured material attached to the Perfecta droplets. In d) the pure Perfecta nano particles show no special features.

To gain further insight into self‐assembly of these samples, cryo‐TEM measurements were performed. Representative images are shown in Figures 3a–c and S3 for cubosome samples, prepared at different PERFECTA concentrations, and F‐127 NPs at 5 mg/mL of PERFECTA (Figure 3d). The images show that PERFECTA preferentially segregates into spherical domains, which are either embedded within the cubosomes (white arrows, see also Figure S4 for more details), or not well‐structured NPs which can be interpreted as intermediate state of cubosomes (Figure 3b, double black arrows), or present as isolated NPs, some of which have a surrounding layer. Both embedded and isolated PERFECTA spherical domains were approximately 100 nm in size. F‐127 PERFECTA NPs displayed a similar morphology to those associated to cubosomes, however, they were larger, significantly more polydisperse and no surrounding layer could be observed. To elucidate the formation mechanism of cubosomes containing embedding fluorinated spherical domains formed by PERFECTA, we performed DLS and SAXS analyses at different stages of the preparation procedure: 1) immediately after sonication of the biphasic mixture and 2) following evaporation of the organic solvent. These studies revealed the formation of amorphous NPs with a size of approximately 255 nm and low polydispersity (0.1) immediately after sonication, as indicated by the absence of crystalline peaks in the SAXS profile. In contrast, the characteristic *Pn3m* cubic liquid‐crystalline lattice emerged only after solvent evaporation, while NPs size and PDI remained almost unchanged (236 nm, polydispersity 0.1, see Figure S5). Besides, cryo‐TEM images taken at different sonication conditions allow us to conclude that sonication at 70°C is essential to achieve homogeneous mixing of PERFECTA and phytantriol. Indeed, when sonication was performed at temperatures between 25°C and 37°C during 30 minutes, visible precipitate formed in the samples, and the resulting dispersed NPs were smaller than those obtained at 70°C, with a reduced encapsulation efficiency (hydrodynamic radius: 180 nm, EE: 55%). Cryo‐TEM images of these samples revealed the presence of very large, well‐ordered cubic NPs without spherical PERFECTA domains, as well as small nanoassemblies containing PERFECTA domains and a surrounding layer, likely representing early stages of cubosome formation. Additionally, many poorly structured NPs, similar to those occasionally observed in Figure 3b, were present (see Figure S6). Moreover, at 70°C, longer sonication times (30 min vs. 5 min) resulted in fewer non‐structured NPs and lower amount of PERFECTA (Figure S7).

These observations point to two possible scenarios. First, PERFECTA may rapidly segregate into spherical domains stabilized by polymer and/or lipid coatings to minimize its exposure to the aqueous environment; these domains could subsequently interact and fuse with the phytantriol cubic phase. Alternatively, PERFECTA may initially mix with phytantriol during the high‐temperature sonication process in the presence of ethyl acetate. Upon solvent evaporation, the formation of the liquid crystalline phase could then drive the segregation of excess PERFECTA into attached spherical domains, some of which may detach to yield the isolated PERFECTA NPs observed in cryo‐TEM images. To discriminate between these hypotheses, we investigated the order–disorder phase transitions of cubosomes as a function of temperature—a hallmark of liquid crystalline systems—in order to assess the incorporation of PERFECTA within the phytantriol bilayer.

SAXS experiments were therefore performed by heating PERFECTA loaded cubosomes, at different concentrations, from 25°C in a temperature range between 50°C and 70°C (see Figure S8a–d). Cubosomes, both empty and loaded with 1 mg/ml of PERFECTA, retained their internal crystalline structure at all investigated temperatures, although a shift of the primary diffraction peaks toward higher q values (approximately 0.15 and 0.19 Å^−1^ for the first two reflections) was observed, consistent with a decrease of cell parameter. In contrast, formulations containing higher PERFECTA concentrations exhibited a loss of long‐range order at 70°C, indicative of a transition to a disordered phase (see Figure S8). These results support the hypothesis that PERFECTA is at least partially incorporated into the lipid bilayer, where its presence disturbs the internal liquid‐crystalline order promoting the transition to a disordered structure at lower temperatures with respect to the pristine cubosomes.

**FIGURE 4:**
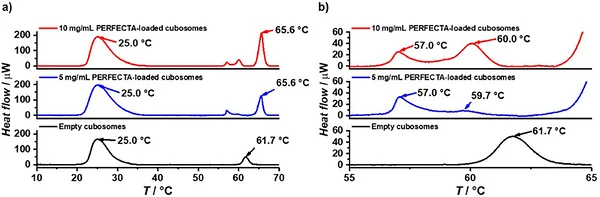
DSC curves of the indicated samples. (a) Full heat‐flow profiles showing the endothermic peaks attributed to Pluronic F‐127 association (25°C), cubosome order–disorder transition, and PERFECTA thermal transition (65.6°C). (b) Magnified view of the 55°C–65°C region highlighting shift to lower temperature and peak splitting of the cubosome order–disorder transition upon PERFECTA loading. Peak temperatures are indicated.

Notably, the broad peak characteristic of the L2 disordered phase, previously observed in phytantriol liquid crystalline NPs above the transition temperature, is absent in PERFECTA‐loaded samples. This suggests that, under these conditions, the system does not adopt the reverse micellar phase with aqueous domains, but rather phytantriol‐PERFECTA NP might be formed with expulsion of water. To further corroborate these findings and quantitatively assess the dependence of the transition temperature on PERFECTA loading, differential scanning calorimetry (DSC) experiments were conducted (Figure 4). Indeed, DSC has been widely applied in pharmaceutical and lipid‐based systems to investigate how incorporated guest molecules modify lipid phase behavior. Although it is more commonly used for lamellar liposomes, it provides useful and more detailed information on how the phase transition of a lipid‐based system is affected by the presence of guest molecules [19, 20]. In empty cubosomes, two distinct peaks were observed: the first, at 25°C, attributed to the temperature‐induced association of Pluronic F‐127; [21, 22] the second, at 61.7°C, corresponding to a cooperative order‐disorder transition of cubosomes in the excess of water [22]. PERFECTA‐loaded cubosomes displayed three transition events at approximately 57°C, 60°C, and 65.6°C. The latter peak is consistent with a thermal event previously reported for PERFECTA itself in lipid NPs [23], confirming that a fraction of the probe is incorporated within the bilayer. The two remaining peaks at 57°C and 60°C are both shifted to lower temperatures relative to the pure cubosome transition at 61.7°C, supporting SAXS data. The appearance of two order‐to‐disorder transitions suggests a non‐uniform distribution of PERFECTA within the phytantriol bilayer, implying the coexistence of regions with distinct local environments. PERFECTA‐rich regions likely undergo the transition at lower temperatures due to local perturbations in lipid packing and curvature stress, whereas PERFECTA‐poor regions retain a transition temperature closer to that of the pristine phytantriol cubosomes. Overall, DSC results further confirm that a fraction of PERFECTA is incorporated within the phytantriol lipid bilayer of the cubosomes. This experimental evidence makes the second hypothesis, for example, segregation of PERFECTA domain subsequent to expulsion through cooling down during preparation, the most reliable, as the insertion of PERFECTA in the lipid bilayer most probably happens at high temperature during cubosome formation. However, further investigations would be required to unambiguously resolve the spatial organization of PERFECTA within the membrane.

**FIGURE 5:**
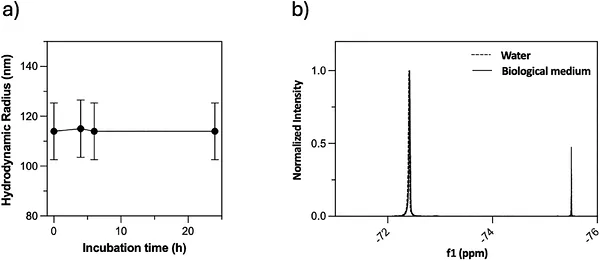
(a) Mean hydrodynamic radius measured at 90° scattering angle of 5 mg/ml PERFECTA loaded cubosomes incubated in DMEM + 10% FBS measured at different time points within 24 h. (b) ^19^F‐NMR comparison of 5 mg/ml PERFECTA loaded cubosomes in water and after 24 h of incubation in DMEM + 10% FBS. PERFECTA peak at around −72.4 ppm, TFA reference set at −75.5 ppm. Colour codes as reported in figure.

Overall, the obtained PERFECTA loaded cubosomes were characterized by good colloidal stability and high encapsulation efficiencies across different PERFECTA loadings. Knowing that the cubic phase of PERFECTA loaded cubosomes was retained until 50°C for all samples (see Figure S8), with a view toward future preclinical applications, we evaluated the colloidal stability of PERFECTA‐loaded cubosomes in biologically relevant media. To this end, cubosomes containing 5 mg/mL PERFECTA were incubated in cell culture medium supplemented with 10% fetal bovine serum (FBS), mimicking in vitro cell culture conditions. The dispersions were maintained at 37°C and 95% relative humidity for 24 h, after which their stability was assessed by DLS, SAXS, and ^19^F‐NMR analyses. DLS measurements indicated that NPs remained detectable in the biological medium after 24 h, with hydrodynamic diameters remaining essentially unchanged over time (Figure 5a). SAXS analysis further confirmed the preservation of the cubic liquid‐crystalline phase after 3 and 12 h of incubation, as evidenced by the characteristic diffraction peaks. After 24 h, however, the SAXS profiles became noisier, making the persistence of long‐range cubic order less conclusive (Figure S9). Finally, ^19^F‐NMR analysis of 5 mg/mL PERFECTA‐loaded cubosomes dispersed in water and in biological medium revealed that the fluorinated cargo was retained even after 24 h of incubation. As shown in Figure 5b, the ^19^F signal corresponding to PERFECTA (−72.4 ppm) remained unchanged relative to the TFA reference peak (−75.5 ppm), indicating a preserved fluorine content equivalent to 85.5% of the initial PERFECTA mass. These findings provide strong support for the stability of the system under biologically relevant conditions and are essential for its prospective biomedical applications.

**FIGURE 6:**
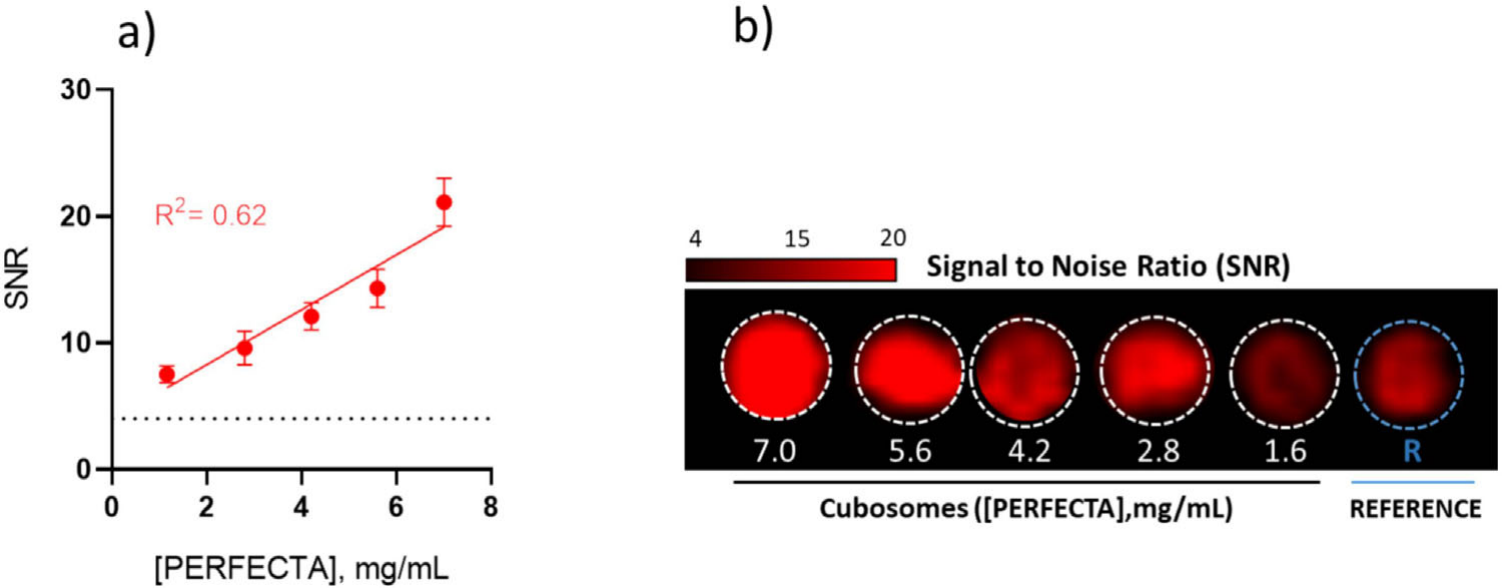
(a) ^19^F‐MRI signal to noise ratio of PERFECTA loaded cubosomes as a function of PERFECTA concentration. (b) Phantom MRI images of serial dilutions of PERFECTA loaded cubosomes (1.6–7.0 mg/mL PERFECTA) R = reference sample consisting of a PERFECTA–lecithin emulsion with a known PERFECTA concentration of 2.3 mg/mL.

Finally, relaxometric properties of PERFECTA loaded cubosomes were investigated to assess their suitability for ^19^F‐MRI. ^19^F‐NMR measurements yielded longitudinal (*T*_1_) and transverse (*T*_2_) relaxation times of 497 ± 50 ms and 281 ± 4 ms, respectively. These values are comparable to those reported for other established PERFECTA‐based formulations, such as lecithin emulsions and polymer stabilized NPs [14], which have previously demonstrated optimal performance as ^19^F‐MRI nanoprobes [18]. Importantly, these relaxometric parameters indicate that the PERFECTA‐loaded cubosomes possess favourable properties for ^19^F‐MRI, enabling the use of fast imaging sequences. This is further supported by the robust^19^F MRI signal observed in the phantom studies at varying PERFECTA concentrations (Figure 6), where the signal intensity remained above the detection threshold for PERFECTA concentrations ≥ 1.6 mg/mL.

## 4 Conclusion

In this work, we demonstrated the development of PERFECTA loaded LCNPs as ^19^F‐MRI nanoprobes. Specifically, phytantriol was employed as lipid matrix and Pluronic F‐127 as a steric stabilizer to produce stable and monodisperse cubosomes exhibiting an internal liquid crystalline order with *Pn3m* symmetry. The resulting cubosomes showed a high encapsulation efficiency, exceeding 70% of the initial PERFECTA content, with final probe concentrations ranging from 1 to 10 mg/mL. The stability of the LCNPs in both aqueous and biological media was confirmed by DLS and SAXS analyses, while ^19^F‐NMR enabled quantification of the encapsulated fluorine. Furthermore, ^19^F‐MRI phantom studies demonstrated the suitability of these NPs for bioimaging applications. Cryo‐TEM imaging revealed that part of PERFECTA tends to segregate into spherical domains located at the surface of the cubosomes. These domains appear to be, in most cases, embedded within the LCNP, likely due to the presence of the stabilizing polymer. This observation is supported by control samples containing only F‐127, which exhibited similar spherical NPs with a ^19^F‐NMR chemical shift overlapping those of PERFECTA‐loaded cubosomes. Nevertheless, SAXS measurements highlighted a concentration‐dependent effect of PERFECTA on the internal liquid crystalline organization upon increasing temperature. In particular, at 70°C, cubosomes loaded with 5 and 10 mg/mL PERFECTA exhibited a transition to a disordered phase, unlike empty cubosomes or those loaded with 1 mg/mL. These findings suggest that a fraction of PERFECTA is also incorporated within the phytantriol lipid bilayer, albeit in amounts insufficient to significantly alter the structural parameters. This interpretation is further supported by micro‐DSC analysis, which showed that the presence of PERFECTA lowers the temperature at which the order–disorder transition occurs. Overall, these results provide valuable insights into the interaction between fluorinated compounds and lipid liquid crystalline phases, from both a fundamental and applied perspective. The presence of internal aqueous channels within the cubosomes also offers opportunities for the co‐encapsulation of hydrophilic cargos, such as nucleic acids. Combined with the efficient incorporation of PERFECTA, this platform could represent a promising nanotheranostic system for future biomedical applications.

## Conflicts of Interest

The authors declare no conflicts of interest.

## Supporting information



The authors have cited additional references within the Supporting Information.

## Data Availability

The data that support the findings of this study are available from the corresponding author upon reasonable request.
