# Supplementary material for: PERFECTA Loaded Lipid Cubosomes for 19F‐MRI: Physico‐Chemical Characterization
Source: Chemistry. 2026 Jun 8;32(35):e71231. doi: 10.1002/chem.71231 (PMC13587639; doi:10.1002/chem.71231)
Supplement: Supplementary file 1 [file CHEM-32-e71231-s001.docx]

**Supplementary Information**

**PERFECTA loaded lipid cubosomes for ^19^F-MRI: Physico-chemical characterization**

Pietro Milesi ^[a]^, Beatrice Lucia Bona^[a]^, Cristina Chirizzi^[b]^, Marco Campanile^[d]^, Pompea Del Vecchio^[d]^, Marc Schmutz^[e]^, Valerie Sartor^[c]^, Debora Berti^[f]^, Pierangelo Metrangolo^[a]^,Francesca Baldelli Bombelli^[a]^*, Barbara Lonetti^[c]*^

**Figure S1**. **a)** Small angle X ray intensity as a function of scattering vector for phytantriol based cubosomes obtained with emulsification protocol; **b)** typical phytantriol cubosomes obtained through the described emulsification protocol

| Peak Position | Miller indices (hkl) | Normalized Ratio of Brag Peak positions |
| --- | --- | --- |
| 0.132 Å | 110 | 1 |
| 0.162 Å | 111 | 1.23 |
| 0.186 Å | 200 | 1.41 |
| 0.228 Å | 211 | 1.73 |

**Table S1.** Values of the peak positions and peak assignment

**Figure S2**. DLS results: comparison of PERFECTA loaded cubosomes stored in water and measured right after their formulations and after 2 weeks. Hydrodynamic radius profiles were obtained at T=90° measuring scattering angle, as an average of three different measurements.

| Formulation Type | PERFECTA Concentration  (mg/ml) | Hydrodynamic Diameter  (nm, SD) | Polydispersity Index  (SD) | PERFECTA Encapsulation Efficiency  (%, SD) |
| --- | --- | --- | --- | --- |
| F-127 NPs | 0 | 390 (70) | 0.26 (0.14) | - |
| F-127 NPs | 1 | 136 (55) | 0.51 (0.01) | 64 (21) |
| F-127 NPs | 5 | 229 (35) | 0.31 (0.01) | 70 (5) |
| F-127 NPs | 10 | 205 (18) | 0.23 (0.06) | 71 (1) |

**Table S2.** Comparison of formulations of PERFECTA loaded F-127 Pluronic NPs, obtained using different PERFECTA concentrations of ethyl acetate solution. The hydrodynamic diameter was evaluated through DLS, the encapsulation efficiency with ^19^F NMR. SD provided as a means of at least three different measurements, obtained at different measuring angles.

**a)	b)	c)**

**g)	h)	i)**

**d)	e)	f)**

**l)	m)	n)**

**Figure S3**. Cryo-TEM images of phytantriol cubosomes stabilized by F127 prepared at different PERFECTA concentrations: (a-c) 1 mg/ml; (d-f) 5 mg/ml; (g-i) 10 mg/ml. Cryo-TEM image of F127 stabilized PERFECTA NPs prepared at 10 mg/ml of PERFECTA (l-n).

**FigureS4**. Images taken at 40°, 0°and -20° degrees of tilt are shown.The white and black arrows point to cubosomes where Perfecta is encapsulated. By tilting, the overall shape of the cubosome is clearly changed to the one pointed by the white arrow. In that case, the cubosome is out of the tilting axis, and this clearly demonstrates that the Perfecta sphere is encapsulated in the cubic phase. The long black arrow points to the supporting film which is damaged by the beam.

**FigureS5**. (a) Intensity weighted hydrodynamic radius distribution obtained by DLS analysis of typical PERFECTA loaded cubosomes before and after solvent evaporation using rotary evaporation. Hydrodynamic radius profiles obtained at 90° scattering angle as average of three different measurements. (b) SAXS intensity as a function of the scattering vector of typical PERFECTA loaded cubosomes measured before and after ethyl acetate removal. Color codes as reported in figure.

**Figure S6.** Cryo-TEM images for sample prepared with 30 min sonication between 25°C and 37°C. We can observe a lot of small Perfecta nanoparticles with some material surrounding them.

**Figure S7.** Cryo-TEM images for sample prepared with 5 min sonication at 70°C. Smaller cubosomes are observed and the Perfecta NPs are visible. They are surrounded or not by non structured material.

a)	b)

c)	d)

e)

**Figure S8.** SAXS intensity as a function of the scattering vector, relative to a) empty phytantriol cubosomes at different temperatures; b) 1mg/ml PERFECTA loaded cubosomes at different temperatures; c) 5 mg/ml PERFECTA loaded cubosomes at different temperatures, d) 10 mg/ml PERFECTA loaded cubosomes at different temperatures; e) comparison between empty and PERFECTA loaded cubosomes after heating from 25 °C until 70 °C.

**Figure S9**. SAXS intensity as a function of the scattering vector, comparison between PERFECTA loaded cubosomes after different incubation times in biological medium
